# Impaired access of lymphocytes to neoplastic prostate tissue is associated with neoangiogenesis in the tumour site

**DOI:** 10.1038/sj.bjc.6603650

**Published:** 2007-02-27

**Authors:** S Fedida, D Fishman, Z Suzlovich, S Argov, M Friger, L Oren, S Segal, N Sion-Vardy

**Affiliations:** 1 Department of Microbiology and Immunology, Faculty of Health Sciences, Ben-Gurion University Cancer Research Center, Ben-Gurion University of the Negev POB 653, Beer-Sheva 84105, Israel; 2 Department of Morphology, Faculty of Health Sciences, Ben-Gurion University Cancer Research Center, Ben-Gurion University of the Negev POB 653, Beer-Sheva 84105, Israel; 3 Institute of Pathology, Soroka University Medical Center, Beer-Sheva, Israel; 4 Department of Epidemiology, Faculty of Health Sciences, Ben-Gurion University Cancer Research Center, Ben-Gurion University of the Negev POB 653, Beer-Sheva 84105, Israel; 5 Department of Behavior Sciences, College of Judea and Samaria, POB 3, Ariel, Israel

**Keywords:** prostatic neoplasms, prostatic hyperplasia, neovascularization pathologic, tumour escape

## Abstract

Recent reports demonstrated that neovasculature of certain murine tumours inhibits migration of lymphocytes to malignant tissues. We examined the possible existence of this phenomenon in human prostate adenocarcinoma by relating extent, patterns and composition of leucocyte infiltrates in adenocarinoma specimens (*N*=28) to microvessel density and percentages of these vessels expressing adhesion molecules CD54, CD106 and CD62E. Specimens of nodular hyperplasia (*N*=30) were used as a control for nonmalignant prostate. Increased microvessel density was detected in foci of adenocarcinoma, as compared with adjacent benign areas (*P*=0.004) or hyperplastic specimens (*P*=0.001). Only CD54 was detected on prostate vasculature; percentages of CD54-expressing vessels in adenocarcinoma lesions and adjacent areas were higher than in hyperplasia (*P*=0.041 and *P*=0.014, respectively). Infiltrating leucocytes were either scattered diffusely in tissue or organised into clusters mainly composed of CD4-positive lymphocytes; smaller percentage of tissue was occupied by clustered infiltrates in adenocarcinoma foci (mean=0.7; median=0; range=0–5) than in adjacent tissue (mean=2.5; median=1; range=0–15; *P*=.021) and hyperplasia (mean=1.9; median=2; range=0–5; *P*=.006). In adenocarcinoma foci, microvessel density tended to negatively correlate with percentage of tissue occupied by an overall leucocyte infiltrate (mean=8.6; median=7.5; range=30) and negatively correlated with percentage of tissue occupied by clustered infiltrate (*P*=0.045). Percentage of CD54-expressing vessels positively correlated with percentage of tissue occupied by an overall (mean=12; median=10; range=30; *P*=0.01) and clustered (*P*=0.023) infiltrate in hyperplasia, whereas in carcinoma-adjacent benign areas, correlation was detected only for clustered infiltrates (*P*=0.02). The results indicate that impaired access of lymphocytes to malignant lesions is associated with increased numbers of newly formed blood vessels, whereas vascular CD54 likely contributes to extravasation of lymphocytes only in benign prostate tissue.

Although many types of malignancies are potentially antigenic and immunogenic (Romero *et al*, 1998; Lee *et al*, 1999; Campoli *et al*, 2005), immune-effector cells recognising tumour-specific antigens fail to reject the tumour, when grafted into the host circulation (Prévost-Bondel *et al* 1998; Romero *et al*, 1998; Lee *et al*, 1999). Neoangiogenesis is an integral part of tumour progression, where a vasculature in primary neoplastic lesion undergoes ‘angiogenic switch’ and converts into a chaotic network of rapidly proliferating blood vessels displaying marked structural and molecular abnormalities (Bono *et al*, 2002; Ryschich *et al*, 2002; Strohmeyer *et al*, 2004; Ozawa *et al*, 2005). Studies employing experimental murine tumour microcirculation models and intravital fluorescent microscopy techniques demonstrated impaired interactions between circulating leucocytes and endothelial cells, thus implying the existence of a barrier function in tumour-associated blood vessels (Ryschich *et al*, 2002; Dirkx *et al*, 2003; Garbi *et al*, 2004). Of particular interest are reports explaining the ‘barrier’ effect of tumour neovasculature by diminished expression of cytoadherence molecules (CAMs) on endothelial lining of blood vessels (Tromp *et al*, 2002; Dirkx *et al*, 2003, 2006; Bouma-ter Steege *et al*, 2004; Baluk *et al*, 2005). For example, more intense infiltration of malignant lesions by lymphocytes was observed in specimens of medullary breast carcinoma displaying elevated expression of CD54 (ICAM-1) and CD106 (VCAM-1) as compared with ductal breast carcinoma displaying diminished expression of these CAMs (Bouma-ter Steege *et al*, 2004). Correspondingly, the experiments on tumour-bearing mice demonstrated upregulation of vascular CAMs expression following angiostatic therapy or of T-cell therapy combined with trigger of inflammation, which was accompanied by intense infiltration of malignant lesions by lymphocytes and reduction of tumour size (Garbi *et al*, 2004; Dirkx *et al*, 2006). Despite the obvious attractiveness of mode of tumour-immune evasion, it cannot be automatically applied to all types of murine and human malignancies because of marked differences between vascular beds in distinct anatomic locations (Essler and Ruoslahti, 2002).

The incidence of prostate carcinoma is increasing steadily and no efficient curative approaches are currently available. In course of this disease, the numbers of newly formed microvessels progressively increase (Montironi *et al*, 1993; Mazzucchelli *et al*, 2000; Bono *et al*, 2002; Strohmeyer *et al*, 2004; Ozawa *et al*, 2005). However, it is not known whether these neovessels may attenuate migration of lymphocytes to malignant lesions and if so, whether vascular CAMs are involved. We address this issue by relating microvessel density (MVD) and percentages of these vessels expressing major CAMs to the extent and patterns of leucocyte infiltrate in specimens of human prostate adenocarcinoma. Because nodular hyperplasia of the prostate gland (NHPG) is a common histopathological finding in males of this age group and hyperplastic changes were also detected in perimalignant benign areas of carcinoma specimens, tissue specimens affected by NHPG were used as a control for the prostate gland not affected by the malignant disease.

## MATERIALS AND METHODS

### Tissue specimens

A series of 28 prostate adenocarcinoma specimens obtained by radical prostatectomy and 30 NHPG specimens obtained by transurethral resection were available from the Institute of Pathology, Soroka University Medical Center. Gleason score values for carcinomas were 5 in one out of 28 cases, 6 in four out of 28 cases, 7 in 17 out of 28 cases, 8 in four out of 28 cases and 9 in two out of 28 cases. All tumours were of Stage II T_1c_–T_2_ N_0_ M_0_. The mean age of carcinoma patients was 64 years, mean age of NHPG patients was 71 years. All examinations employing specimens obtained from patients were approved by the institutional Helsinki Committee and were carried out according to the Israeli law.

### Immunohistochemistry, estimation of MVD and assessment of infiltrate

Sections of prostate tissue were reacted to commercial antibodies diluted, as follows: monoclonal anti-CD34 (Dako Cytomation A/S, Glosstrop, Denmark), diluted 1 : 100; monoclonal anti-CD54 (Zymed Laboratories Inc., South San Francisco, CA, USA) ready to use; monoclonal anti-CD106 (Santa Cruz Biotechnology Inc., Santa Cruz, CA, USA), diluted 1 : 25 and 1 : 50; monoclonal anti-CD106 (Dako Cytomation), diluted 1 : 25; monoclonal CD62E (Santa Cruz), diluted 1 : 25; monoclonal antiinterleukin (IL)-10 (R&D Systems Europe Ltd, Abington, UK), diluted 1 : 20; monoclonal anti-CD45 (Dako Cytomation), diluted 1 : 100; anti-CD3 (Dako Cytomation), diluted 1 : 100; anti-CD4 (Zymed) ready to use; anti-CD8 (Dako Cytomation), diluted 1 : 25; anti-CD20 (Dako Cytomation), diluted 1:100; anti-CD14 (Zymed), diluted 1 : 50; and anti-CD15 (Dako Cytomation), diluted 1 : 50. Antigen retrieval procedures included boiling for 20 min in 20 mmol citrate buffer solution (pH 6) (for anti-CD34, anti-CD45, anti-CD3, anti-CD4, anti-CD8, anti-CD20, anti-CD15 anti-IL-10 antibodies), boiling for 20 min in a Tris–EDTA buffer (10 mmol Tris base, 1 mmol EDTA and 0.05% Tween 20 (pH 9)) (for anti-CD106 antibodies), 0.15% trypsin in water (pH 7.8) for 20 min at 37°C (for anti-CD14 antibodies). Sections of human tonsils were used as positive controls for the aforementioned antibodies. To estimate MVD, specimens were screened at × 200 magnification to detect areas enriched with blood vessels (‘hot spots’). Microvessel density was assessed in three ‘hot spots’ by counting blood vessels displaying CD34 immune reactivity at × 400 magnification simultaneously by three observers (NSV, SF and ZS), using a multiple-head light microscope and expressed as a mean of three ‘hot spots’. The counts were assessed in a blind manner without the knowledge of patient's clinical course or outcome. Any single endothelial cell or cluster of endothelial cells positively stained by anti-CD34 antibodies was considered as a microvessel. Vessels of a caliber larger than approximately eight red blood cells were excluded from the analysis. To attribute the expression of CAMs to microvessels, ‘hot spots’ in anti-CD34-probed sections were marked, and areas corresponding to the marked ‘hot spots’ were found in successive tissue sections probed with anti-CAM antibodies (only CD54 was detected in prostate vessels) and used for counting of CD54-expressing microvessels. The percentages of CD54-expressing microvessels were calculated using estimated values of MVD. The quantification of infiltrating leucocytes (assessed according to immune reactivity to CD45 or markers of specific leucocyte subsets) was performed at × 100 magnification simultaneously by three independent observers (SF, ZS and DF) by estimating percentage of tissue occupied by any given component (single cells or cluster of cells).

### Statistical analysis

SPSS software was used for statistical calculations. Microvessel density and percentages of CD54-expressing blood vessels in carcinoma lesions were compared with those in benign perimalignant areas by using paired *t*-test. Microvessel density and percentages of CD54-expressing blood vessels in carcinoma lesions or perimalignant benign zones were compared with those in NHPG by using grouped *t*-test. χ^2^ Test was used to compare IL-10 expression in carcinoma lesions, perimalignant benign areas and NHPG. Correlations between variables were computed by using Pearson's correlation (*r*).

## RESULTS

**Figure 1:**
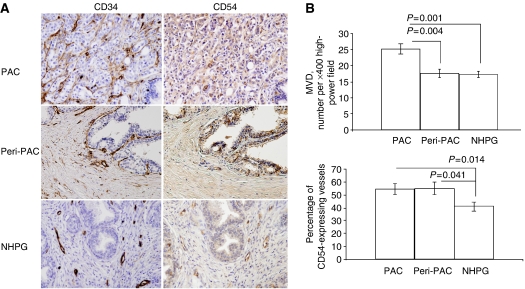
Microvessel density and percentages of CD54-expressing vessels in adenocarcinoma lesions (PAC), adjacent benign tissue (peri-PAC) and specimens of nodular hyperplasia of the prostate gland (NHPG). (**A**) Typical photographs of successive tissue sections reacted to anti-CD34 and anti-CD54 antibodies (magnification × 200). (**B**) Microvessel density and percentages of CD54-expressing vessels in PAC, peri-PAC and NHPG (mean+s.e.m.).

A significantly higher MVD was found in the vicinity of adenocarcinoma lesions (PAC) (25.18+2), as compared with benign perimalignant regions of the same specimens (peri-PAC) (17.7+1.3) (*P*=0.003) and specimens affected by NHPG (17.4+0.9) (*P*=0.001), whereas no difference in MVD values between peri-PAC and NHPG was detected (Figure 1). Among CAMs analysed, only CD54 was expressed by the prostate vasculature (Figure1A and data not shown). We detected higher percentages of CD54-expressing microvessels in PAC and peri-PAC, as compared with NHPG (*P*=0.014 and *P*=0.041, respectively; Figure 1). It is noteworthy that CD54 was also identified on glandular epithelium; however, only CD54-expressing blood vessels were counted.

**Figure 2:**
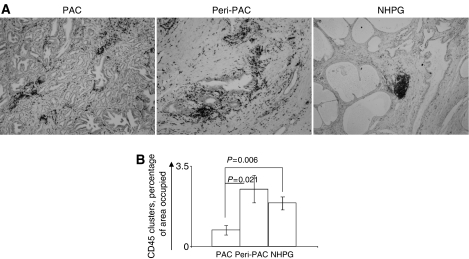
Patterns of leucocyte infiltration in adenocarcinoma lesions (PAC), adjacent benign tissue (peri-PAC) and specimens of nodular hyperplasia of the prostate gland (NHPG). (**A**) Tissue infiltrating leucocytes in PAC, peri-PAC and NHPG identified according to CD45 immune reactivity (magnification × 40). (**B**) Percentages of tissue area occupied by clustered infiltrates assessed according to CD45 immune reactivity in PAC, peri-PAC and NHPG (mean+s.e.m.).

**Figure 3:**
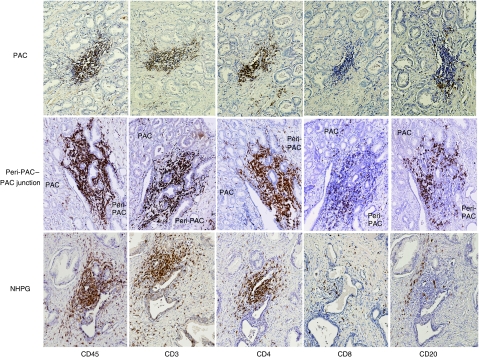
Composition of clustered infiltrates in adenocarcinoma lesions (PAC), adjacent benign tissue (peri-PAC) and specimens of nodular hyperplasia of the prostate gland (NHPG). Successive tissue sections were reacted to antibodies recognising common (CD45) and subset-specific (CD3, CD4, CD8 – T lymphocytes, CD20 – B lymphocytes) markers of leucocytes. Magnification × 200.

Most tissue-infiltrating leucocytes detected according to CD45 immune reactivity resided in periglandular stroma and only few intraepithelial leucocytes were identified. Two distinct patterns of infiltration were detected, that is, diffuse periglandular infiltrates and leucocytes clustered into follicle-like aggregates (Figure 2A). A significantly lower percentage of tissue occupied by clustered infiltrates assessed according to CD45 immune reactivity was found in PAC (mean (M)=0.7; median (Mdn)=0; range (Rng)=0–5; standard error mean (s.e.m.)=0.2), as compared with peri-PAC (M=2.5; Mdn=1; Rng=0–15; s.e.m.=0.7; *P*=0.021) and NHPG (M=1.9; Mdn=2; Rng=0–5; s.e.m.=0.3; *P*=.006) (Figure 2B), whereas no such differences among PAC, peri-PAC and NHPG were demonstrated for percentage of tissue occupied by an overall leucocyte infiltrate (data not shown). Irrespective of their location (PAC, peri-PAC or NHPG), clusters were mainly composed of CD3-positive cells (CD4-positive cells predominated over CD8-positive cells) and less of CD20-positive cells (Figure 3), whereas no CD14- or CD15-positive cells were detected in clusters; the latter cells were scattered in the stromal tissue at very low numbers and not detected in most specimens (data not shown). There was a tendency towards negative correlation between MVD and percentage of tissue occupied by an overall leucocyte infiltrate (diffuse and clustered) assessed according to CD45 immune reactivity in PAC (M=8.6, Mdn=7.5, Rng=0–30, s.e.m.=1) (*r*=−0.338, *P*=0.06), whereas in peri-PAC, a tendency towards positive correlation between MVD and an overall leucocyte infiltrate (M=2.9, Mdn=2, Rng=0–10, s.e.m.=0.58) was detected (*r*=0.304, *P*=0.085) (Table 1). Percentage of tissue occupied by clustered infiltrates assessed according to CD45 and CD4 (M=0.2, Mdn=0, Rng=0–1, s.e.m.=0.01) immune reactivity negatively correlated with MVD in PAC (*r*=−0.362, *P*=0.045 and *r*=0.389, *P*=0.033, respectively); no such correlation was demonstrated in peri-PAC and NHPG (Table 1). However, in NHPG, we detected a positive correlation between percentages of CD54-expressing vessels and percentage of tissue occupied by an overall leucocyte infiltrate assessed according to CD45 (M=12; Mdn=10; Rng=0–30, s.e.m.=1.5) and CD4 (*M*=2.8; Mdn=2; Rng=0–5, s.e.m.=0.3) immune reactivity (*r*=0.454, *P*=0.010 and *r*=0.404, *P*=0.023, respectively). CD54-expressing vessels also correlated with percentage of tissue occupied by clustered infiltrates assessed according to CD45 and CD4 (M=0.9; Mdn=1; Rng=0–3, s.e.m.=0.2) immune reactivity (*r*=0.409, *P*=0.023 and *r*=0.397, *P*=0.024, respectively) (Table 1). In peri-PAC, percentages of CD54-expressing vessels correlated with percentage of tissue occupied by clustered infiltrates assessed according to CD45 immune reactivity (*r*=0.440, *P*=0.020), whereas only a tendency towards positive correlation between CD54-expressing vessels and clustered CD4 infiltrates (*M*=1.1; Mdn=0.5; Rng=0–10, s.e.m.=0.5) was observed (*r*=0.361, *P*=0.054). Neither MVD nor percentages of CD54-expressing vessels correlated with leucocyte infiltrate assessed according to immune reactivity to other leucocyte markers (CD8, CD20, CD14 and CD15) (data not shown).

Many tumours produce inhibitory cytokines (Mantovani *et al*, 2004), which could alter the function of endothelium-adhered leucocytes including their ability to extravasate. Studies performed on murine hepatocellular carcinoma showed that impaired migration of lymphocytes to neoplastic liver tissue coincided with elevated production of IL-10 by endothelial cells of tumour neovessels (Ryschich *et al*, 2006). In this study, we have analysed the expression of IL-10 in 25 of 28 carcinoma specimens and in 29 of 30 NHPG specimens. Interleukin-10 was detected mostly in glandular epithelium in PAC of 14 out of 25 of carcinoma specimens (66%), in peri-PAC of eight out of 25 of carcinoma specimens (33%) and in NHPG of nine out of 29 of NHPG specimens (30%) (Figure 3A and B). No statistically significant correlations between the production of IL-10, vascular expression of CD54 and percentage of tissue occupied by leukcocyte infiltrate were detected (data not shown). None of parameters measured in this study (MVD, percentage of CD54-expressing vessels, clustered infiltrates and IL-10 production) correlated with grade of carcinoma, serum PSA levels and patient's age.

## DISCUSSION

Neoangiogenesis is essential for supplying blood to a rapidly growing neoplastic mass and, therefore, is an integral part of tumour progression. Recent studies performed on experimental murine malignancies ascribed a novel role to newly formed tumour blood vessels, that is, inhibitory effects on proper migration of immune-effector cells to malignant tissue (Ryschich *et al*, 2002; Dirkx *et al*, 2003). However, the relevance of this phenomenon for human cancer is not clear and indirect evidence for endothelial ‘barrier’ effect was provided only for breast and renal cell carcinomas (Griffioen *et al*, 1996; Reiss *et al*, 1998; Bouma-ter Steege *et al*, 2004). Although previous histopathological studies on specimens of human prostate carcinoma showed a relatively rare involvement of malignant glands by lymphocytes (Vesalainen *et al*, 1994; McArdle *et al*, 2004), the possible effects of tumour neovasculature were not considered. To address this issue, we related numbers of newly formed vessels in foci of carcinoma and benign tissue to patterns and extent of leukcocyte infiltrates assessed according to common and subset-specific markers. In concordance with previous reports (Bono *et al*, 2002; Strohmeyer *et al*, 2004), we detected increased numbers of microvessels in the vicinity of malignant glands (Figure 1). Tissue-infiltrating leukcocytes were found in both adenocarcinoma and NHPG specimens; however, the malignant and benign prostate tissues markedly differed in pattern of infiltration (Figure 2). Although leukcocytes scattered diffusely throughout the tissue were identified in foci of adenocarcinoma, adjacent benign tissue and specimens affected by NHPG, leucocytes organised into follicle-like clusters were barely detected in malignant tissue. Because scattered leukcocytes could be found in prostate tissue even in the absence of cancer, hyperplasia or florid prostatitis (McClinton *et al*, 1990; Bostwick *et al*, 2003), we concluded that scattered leucocytes unlikely represent tumour-directed immune-effector cells. On the contrary, the differences in clustered infiltrates between adenocarcinoma lesions, perimalignant tissue and NHPG were not qualitative (irrespective of their location, clusters were mainly composed of CD4 lymphocytes; Figure 3) but rather quantitative (less intense clusters were detected in malignant lesions; Figure 2), thereby implying a diminished access of immune-effector cells to the tumour core. The diminished involvement of malignant lesions by immune cells was associated with augmented neoangiogenesis in the tumour site, as a negative correlation between the extent of clustered infiltrate and values of MVD was detected in foci of carcinoma but not in benign perimalignant areas or NHPG specimens (Table 1). These findings contribute to the hypothesis proposing a novel role for tumour neovasculature in attenuation of lymphocyte extravasation and strongly indicate the existence of endothelial ‘barrier’ phenomenon in human prostate adenocarcinoma.

**Figure 4:**
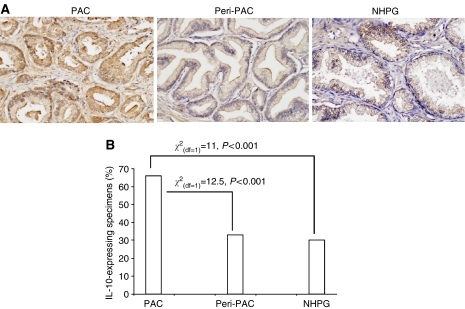
Expression of IL-10 in adenocarcinoma lesions (PAC), adjacent benign tissue (peri-PAC) and specimens of nodular hyperplasia of the prostate gland (NHPG). (**A**) Typical pattern of IL-10 expression in malignant and benign prostate tissue (magnification × 200). (**B**) Percentages of IL-10-expressing specimens.

The molecular mechanism underlying endothelial ‘barrier’ phenomenon is not fully understood. Studies on murine melanona and human breast carcinoma suggest that circulating lymphocytes cannot properly interact with endothelial cells of neovessels expressing diminished levels of CD54, CD106, CD62E and some other CAMs (Dirkx *et al*, 2003; Bouma-ter Steege *et al*, 2004). In our present study, we were able to detect only CD54 (but not CD106 and CD62E) on prostate blood vessels (Figure 1), whereas percentages of CD54-expressing vessels were significantly higher in carcinoma specimens (in both adenocarcinoma foci and perimalignant benign areas) than in NHPG specimens (Figure 1). Intriguingly, a positive correlation between vascular CD54 and percentage of tissue occupied by leucocyte infiltrate was demonstrated only in benign prostate tissue but not in foci of carcinoma (Table 1), thereby implying that this CAM contributes to extravasation of lymphocyte in tissue not affected by malignant transformation. Physiological significance of increased vascular expression of CD54 in prostate carcinoma specimens is not clear. It is noteworthy that, in murine hepatocellular carcinoma, an increased vascular expression of CD54 and CD106 was also observed despite the fact that these neovessels were found to inhibit extravasation of lymphocytes (Ryschich *et al*, 2006). The latter study has also demonstrated that impaired interactions between leukcocytes and vascular endothelium in this tumour coincided with elevated expression of immune-suppressive cytokine IL-10 by endothelial cells. In our study, IL-10 was detected in most of adenocarcinoma lesions (Figure 4); however, we were unable to rely IL-10 expression to leucocyte–endothelial interactions, as functional approaches like intravital microscopy cannot be applied to patients. It is tempting to speculate that circulating lymphocytes are entrapped by CD54-expressing tumour neovessels and become exposed to inhibitory cytokines (e.g. IL-10) and other bioactive substances originating in the tumour site (Mantovani *et al*, 2004).

We cannot exclude a possibility that CD54 expressed by prostate neovasculature is also relevant for processes not directly related to leucocyte–endothelial interactions. Recent reports implicate CD54 to cell signaling cascades involving Src and MAP family kinases and cytoskeletal rearrangements (Wang *et al*, 2003; Kevil *et al*, 2004; Krunkosky and Jarrett, 2006). Therefore, elevated vascular expression of CD54 could be an integral part of tumour-induced ‘angiogenic switch’ characterised by enhanced proliferation and motility of endothelial cells. Further studies are required to test this assumption.

## Figures and Tables

**Table 1:** Correlations between microvessel density (MVD), percentages of CD54-expressing vessels and percentages of tissue occupied by overall and clustered infiltrate assessed according to CD45 and CD4 immune reactivity in malignant lesions (PAC), peri-malignant benign areas (peri-PAC) and nodular hyperplasia of the prostate gland (NHPG)

	**PAC**				**Peri-PAC**				**NHPG**			
	**CD45 total**	**CD45 clusters**	**CD4 total**	**CD4 clusters**	**CD45 total**	**CD45 clusters**	**CD4 total**	**CD4 clusters**	**CD45 total**	**CD45 clusters**	**CD4 total**	**CD4 clusters**
												
MVD	*r*=−0.338	*r*=−0.362*	*r*=−0.159	*r*=−0.389*	*r*=0.304	*R*=0.010	*r*=0.054	*r*=−0.203	*r*=−0.136	*r*=0.156	*r*=−0.122	*r*=−0.031
	*P*=0.06	*P*=0.045	*P*=0.240	*P*=0.033	*P*=0.085	*P*=0.482	*P*=0.408	*P*=0.188	*P*=0.253	*P*=0.234	*P*=0.280	*P*=0.441
												
CD54-expressing vessels	*r*=0.136	*r*=0.064	*r*=−0.024	*r*=0.162	*R*=0.202	*R*=0.440*	*r*=−0.062	*R*=0.361	*r*=0.454**	*R*=0.409*	*R*=0.404*	*R*=0.397*
	*P*=0.274	*P*=0.386	*P*=0.457	*P*=0.230	*P*=0.183	*P*=0.020	*P*=0.395	*P*=0.054	*P*=0.010	*P*=0.023	*P*=0.023	*P*=0.024

* Correlation is significant at 0.05 level (single-tailed test is applied).

** Correlation is significant at 0.01 level (single-tailed test is applied).
